# Esophageal button battery impactions in children: an analysis of 89 cases

**DOI:** 10.1186/s12887-024-04869-x

**Published:** 2024-06-08

**Authors:** Guo Xu, Desheng Jia, Jing Chen, Hongguang Pan, Zebin Wu

**Affiliations:** Department of Otorhinolaryngology, Shenzhen Children’s Hospital, 7019 Yitian Road, Futian District, Shenzhen, Guangdong 518038 China

**Keywords:** Children, Button battery, Esophageal disease, Foreign body, Treatment outcome

## Abstract

**Objective:**

To analyze the clinical characteristics of esophageal button battery impactions in children and explore safe and effective treatment methods.

**Methods:**

This retrospective cohort study was conducted at a single tertiary care center, Shenzhen Children’s Hospital, encompassing 89 children diagnosed with esophageal button battery impactions between January 2013 and January 2023. To minimize esophageal mucosal corrosion, prompt removal of the button battery with a first-aid fast track rigid esophagoscopy under general anesthesia was performed within thirty minutes of diagnosis. The clinical features and complications were recorded and analyzed.

**Results:**

Button battery as esophageal foreign body was prevalent among children under 3 years old (79.8%), with boys exhibiting a higher incidence rate (56.2%) compared to girls (43.8%), and an average age of 25.8 months. The median duration from ingestion to hospital admission was 3 h (range: 0.5 h to 3 months). Common symptoms included vomiting and dysphagia, with early stage vomiting of brown foamy secretions being a characteristic presentation of esophageal button battery impactions. The majority (77.5%) of batteries were lodged in the upper esophagus. The larger batteries were verified to be more prone to complications. All 89 cases exhibited varying degrees of esophageal mucosal erosion, with 31 cases (34.8%) experiencing severe complications, including esophageal stenosis in 11 cases (35.5%), esophageal perforation in 9 cases (29%) with 4 cases of tracheoesophageal fistula, vocal cord paralysis in 6 cases (19.4%), hemorrhage in 2 cases (6.5%), mediastinitis in 2 cases (6.5%), and periesophageal abscess in 1 case (3.2%). Despite the severity of these complications, none of the patients died after emergency surgery.

**Conclusion:**

Esophageal button battery impactions can lead to significant damage to the esophageal mucosa due to its strong corrosiveness. Prompt action is crucial to mitigate the risk of complications. For the first time, we implement a first-aid fast track surgical intervention following diagnosis is imperative to minimize the incidence of adverse outcomes.

## Introduction

Esophageal foreign body (EFB) incidents in children are frequently encountered by pediatric otolaryngologists and often require urgent intervention. While coins, toys, and bones are commonly ingested foreign objects in children and typically do not lead to serious complications [1], the rising prevalence of button battery (BB) ingestion in electronic devices has become a growing concern. BB impactions can result in chemical corrosion, electrical damage, thermal burns, and mechanical compression of the esophageal mucosa. Severe complications, including esophageal perforation, stenosis, tracheoesophageal fistula, and significant hemorrhage, have the potential to be life-threatening [2]. A cohort study revealed a 6.7-fold increase in the relative risk of major or fatal effects of battery-ingestion from 1985 to 2009, as reported by the National Poison Data System [3]. Considering these alarming trends, our study aims to analyze the clinical characteristics of esophageal button battery impactions in the Shenzhen area. We seek to offer valuable insights and guidance for the clinical diagnosis and treatment, ultimately contributing to the improvement of pediatric care and patient outcomes.

## Patients and methods

### Data collection

We retrospectively reviewed a total of 89 children diagnosed with esophageal button battery impactions at Shenzhen Children’s Hospital between January 2013 and January 2023. As the sole tertiary pediatric hospital in Guangdong Province, serving a population of nearly 25 million children, our study encompasses a diverse patient demographic. Notably, the incidence of button battery ingestion is elevated in Guangdong Province, known as the epicenter of the world’s largest electronic commodity market.

Cases in which button batteries were lodged in the nasal passages, oral cavity, pharynx, or the digestive tract below the esophagus were excluded from our study. Clinical data and demographic characteristics, including gender, age, presenting symptoms, preoperative examination findings, battery location and duration of retention, therapeutic outcomes, and complications, were meticulously collected and analyzed. Severe complications were defined as esophageal perforation or stenosis, periesophageal abscess, mediastinitis, tracheoesophageal fistula, vocal cord paralysis, pneumothorax, massive bleeding, or death. Our study adhered to the principles outlined in the 1964 Helsinki Declaration and received approval from the Ethics Committee of Shenzhen Children’s Hospital (protocol number 202,202,602). Written informed consent was obtained from the parent or guardian of each child involved in the study.

### Treatment strategy

Children who were witnessed or suspected of BB ingestion received immediate frontal and lateral X-ray imaging of the neck, chest, and abdomen. Following confirmation of diagnosis, a first-aid fast track rigid esophagoscopy under endotracheal intubation anesthesia was performed within thirty minutes, without the need to wait for preoperative examinations or fasting time. Removal of the button battery utilizes various instruments, including grasping forceps, rat-toothed forceps, tripod forceps, alligator forceps, and retrieval baskets. Endoscopy specifications adhere to those of Karl Storz: < 2 years (0.6*1 cm), 3–5 years (0.7*1.0 cm), 6–10 years (0.8*1.1 cm), and > 11 years (0.9*1.3 cm). Post-removal, copious amounts of normal saline were used to cleanse the esophageal mucosa. Gastric tube feeding was initiated in all, accompanied by acid-inhibiting agents. Antibiotics were utilized to mitigate periesophageal inflammation in cases of severe damage, perforation, or fever. Esophageal imaging (CT or MRI) inspection is performed 1 week after BB removal in patients with significant injuries to confirm the tissue integrity between the esophagus and great vessels.

### Statistical analysis

The data are presented as percentages (categorical variables) and as mean ± standard deviation (continuous variables). Statistical analysis was performed using SPSS software version 26.0.

## Results

### Demographic data

**Fig. 1:**
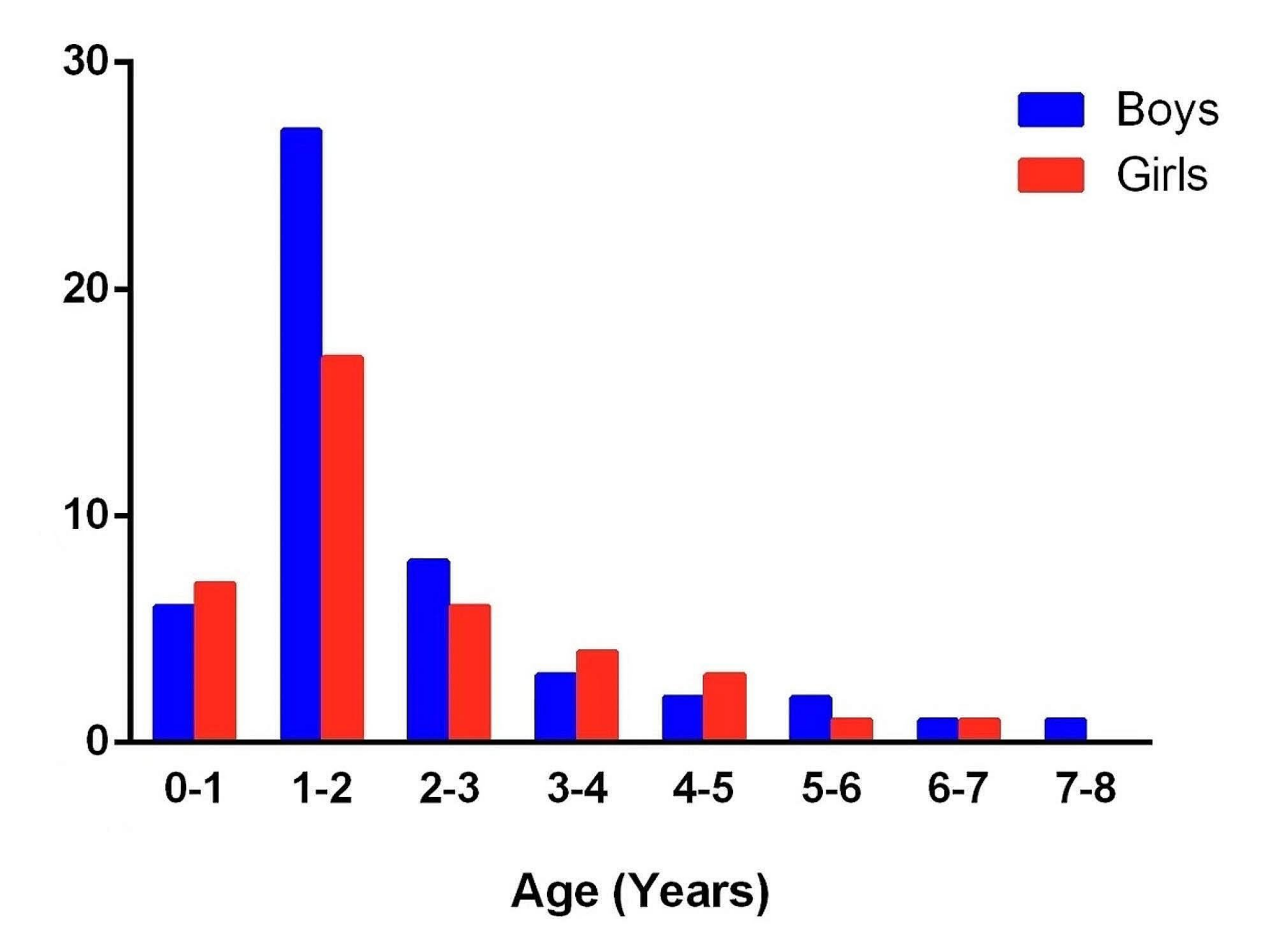
Distribution of pediatric patients by gender and age

89 cases of esophageal button battery impactions were enrolled. The average age of the patients was 2.15 years, ranging from 7 months to 8 years (Fig. 1). 78 cases (87.6%) occurred in children aged 0 to 4 years, with a peak incidence observed between 1 and 2 years (49.4%). Among the cases, there were 50 boys (56.2%) and 39 girls (43.8%), with one patient having Down’s syndrome and another having autism.

### Clinical characteristics

The summary of the clinical characteristics of esophageal button battery impactions cases as presented in Table 1. 72 patients (80.9%) sought medical attention within 24 h of ingestion. The median time from ingestion to hospital admission was 3 h (0.5 h to 3 months). The upper esophagus (above T2 level) was the most common site of impaction, accounting for 69 cases (77.5%), followed by 16 cases (18%) in the middle esophagus, and 4 cases (4.5%) in the lower esophagus. The majority of BBs measured greater than 2 cm in diameter (72 cases, 80.9%), with smaller BBs less common (17 cases, 19.1%). A history of witnessed BB ingestion was documented in 75 cases (84.3%), while the remaining cases were suspected based on symptoms. Patients presented with various symptoms, with vomiting being the most common (57.3%), including vomiting brown foamy secretions in the early stage, followed by dysphagia (41.6%), pain (21.3%), drooling (19.1%), cough (14.6%), and fever (12.4%). Rare symptoms included unexplained crying in 6 cases, hoarseness in 6 cases, laryngeal stridor with dyspnea in 4 cases, and melena in 2 cases. Additionally, 3 cases presented with no obvious discomfort.

**Table 1:** Demographic data, clinical characteristics and analysis of severe complications

Patient characteristics	Numbers (%)	Severe complications		χ2	*P*	
		Yes (*n* = 31)	No (*n* = 58)			
Gender						
Male	50(56.2%)	17	33	0.034	0.852	
Female	39(43.8%)	14	25			
Age						
0-2y	57(64%)	21	36	3.908	0.142	
2-4y	21(23.6%)	9	12			
≥ 4y	11(12.4%)	1	10			
Duration time						
<24 h	70(78.7%)	19	51	8.539	0.003	
≥ 24 h	19(21.3%)	12	7			
Location						
Upper esophagus	69(77.5%)	27	42	3.381	0.184	
Middle esophagus	16(18%)	4	12			
Lower esophagus	4(4.5%)	0	4			
Diameter						
≥ 2 cm	72(80.9%)	20	52	8.262	0.004	
<2 cm	17(19.1%)	11	6			
Witness ingestion						
Yes	75(84.3%)	22	53	6.349	0.012	
no	14(15.7%)	9	5			
Symptoms						
Vomiting	51(57.3%)	20	31	11.284	0.046	
Dysphagia	37(41.6%)	16	21			
Pain	19(21.3%)	9	10			
Drooling	17(19.1%)	8	9			
Cough	13(14.6%)	10	3			
Fever	11(12.4%)	9	2			

Upper esophagus: above T2 level; Middle esophagus: T3-T6 level; Lower esophagus: below T7 level.

### Complications

All 89 cases experienced esophageal mucosal erosion to varying degrees. 31 cases (34.8%) present with severe complications, including esophageal stenosis in 11 cases (35.5%), esophageal perforation in 9 cases (29%) with 4 cases of tracheoesophageal fistula, vocal cord paralysis in 6 cases (19.4%), hemorrhage in 2 cases (6.5%), mediastinitis in 2 cases (6.5%), and periesophageal abscess in 1 case (3.2%). While none of the patients died after emergency surgery.

Our study identified several risk factors significantly associated with complications, including prolonged duration of foreign body impactions, larger diameter of the BB, and lack of witnessed ingestion by a guardian (*P* < 0.05). However, there was no significant difference in the incidence of complications based on gender, age, or location of the foreign body.

**Fig. 2:**
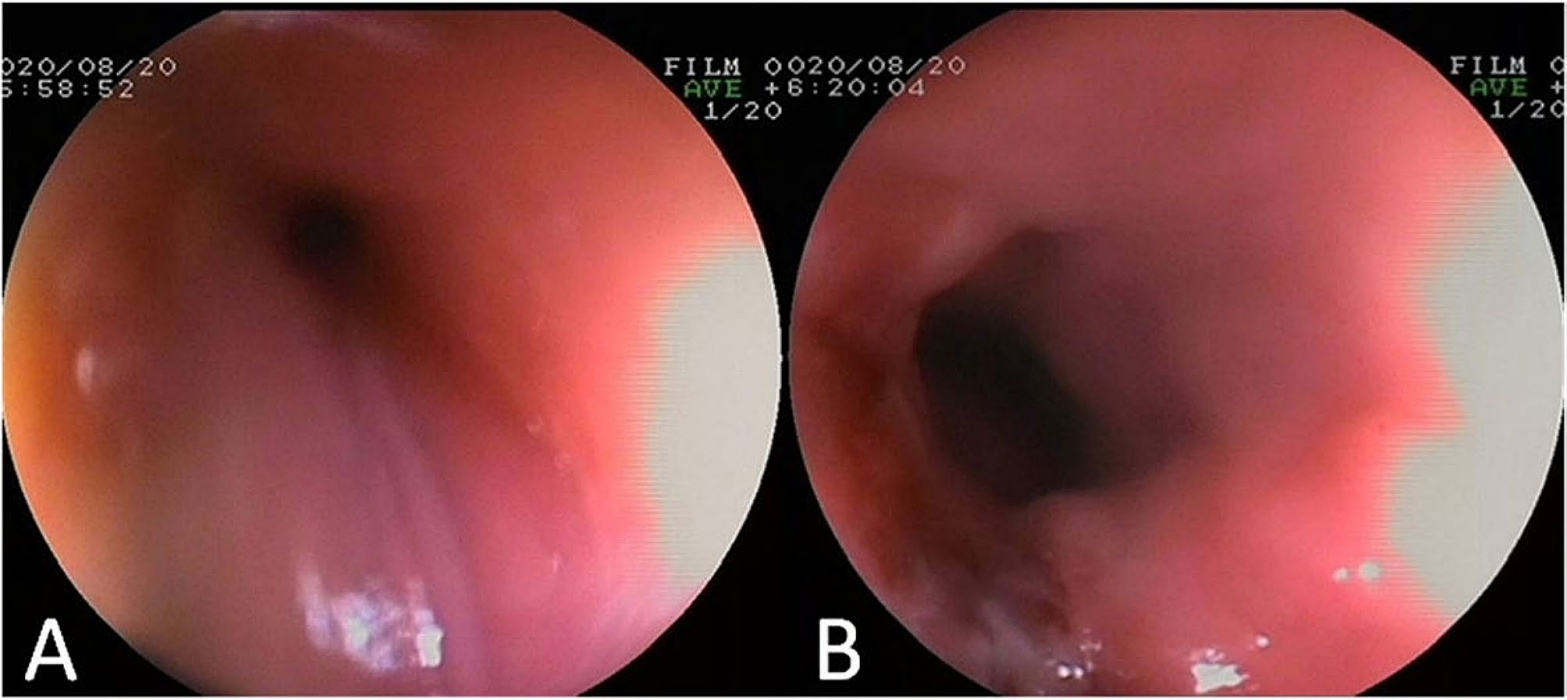
Gastroscopy results (**A**: Oesophageal stenosis, **B**: Patency after dilatation)

Patients with complications were provided with post-operative gastric tube feeding, along with active anti-infection, acid inhibition, and symptomatic treatment for one week. In cases where esophageal stenosis, perforation, vocal cord paralysis, infection, or hemorrhage were observed, MRI and esophageal barium meal examinations were conducted to evaluate the extent of damage to nearby large vessels and airway structures. Reexamination results indicated normal findings in 28 cases with complications following continuous non-operative treatment. However, two cases developed complications of esophageal cicatricial stenosis, manisfesting as difficulty swallowing and only being able to consume liquid, which were successfully treated with balloon esophageal dilatation (Fig. 2) within one month after BB removal. Furthermore, one case developed tracheoesophageal fistula, experiencing recurrent fever and cough post-surgery, which resolved with the placement of self-expandable metallic stents within two months post-BB removal.

The onset times of each complication were summarized. Two cases were detected with tracheoesophageal fistula during the operation, while 1 case was identified within 1-week post-operation and another at 1-month post-operation through esophageal imaging inspection. In the 6 cases of vocal cord paralysis exhibiting different degrees of hoarseness, 4 cases were presented before surgery, while 2 cases developed on the first and second day after surgery diagnosed via electronic fiber laryngoscopy. Two children experienced bleeding during the operation, without further bleeding after treatment with hemostatic materials and drugs.

## Discussion

Esophageal foreign body incidents are common occurrences in children. Particularly, young children are prone to accidental ingestion of foreign bodies as they frequently explore objects with their mouths. The increasing prevalence of BB usage in electronic products in recent years has led to a gradual rise in BB ingestion cases [3, 4]. Our data indicate a rise in esophageal button battery impactions from 6 cases in 2013 to 11 cases in 2023. Unlike other types of foreign body ingestion, BB ingestion poses a significant health risk in the pediatric population due to its highly corrosive nature.

Animal experiments have demonstrated three mechanisms of tissue injury resulting from BB insertion [5, 6]:

1. Burn injury to the esophageal mucosa caused by direct electrical current.
2. Direct corrosive injury to the esophageal mucosa due to electrolyte leakage.
3. Mechanical pressure exerted on surrounding tissues by the batteries.

BB impactions is highly prevalent among children under 6 years of age, particularly in those aged 0 to 4 years. Our data, consistent with previous studies [3, 7], indicates that children under 4 years old accounted for 87.6% of cases. The occurrence of age peaks, notably between 1 and 2 years (49.4%), aligns with the physical and psychological characteristics typical of this developmental stage. Regarding sex distribution, previous studies have shown an even distribution [8]. While our study observed a slight male predominance among cases with severe complications (*n* = 17 of 50, 34%), this difference was not statistically significant (*P* = 0.658).

Children who ingest BBs can exhibit a range of symptoms, often leading to misdiagnosis as respiratory or gastrointestinal disorders. Common clinical presentations of BB impactions include vomiting, dysphagia, pain, drooling, cough, and fever, while rare manifestations may include unexplained crying, hoarseness, laryngeal stridor, dyspnea, and melena. Interestingly, some cases may present with no obvious discomfort [9]. Contrary to the European Society of Pediatric Button Battery Ingestion in Childhood guidelines [10], which emphasize drooling and vomiting as major symptoms, our study found that vomiting and dysphagia were predominant complaints. We noticed that vomiting brown foamy secretions in the early stage was a characteristic manifestation of esophageal button battery impactions. Furthermore, the presence of fever or cough may indicate respiratory or systemic inflammatory responses and is associated with a higher likelihood of complications.

Notably, the incidence of complications differed significantly between cases with witnessed and unwitnessed BB ingestion histories in our cohort. Caregivers may be unaware of the dangers or unable to provide a clear history of BB ingestion, highlighting a delay in seeking medical advice. Our data highlight a concerning finding that 17 patients (19.1%) underwent BB removal more than 24 h after presentation. This underscores the critical need for precise triage practices to promptly identify and refer patients at risk of BB impactions. Implementing such practices is essential for improving outcomes and reducing complications in affected children.

Accurate diagnosis of BB ingestion is paramount, and urgent frontal and lateral X-rays, including the neck, chest, and abdomen, play a crucial role in confirming the site of BB impactions. These imaging studies can reveal a distinctive “double ring or halo sign” on frontal films and a “step-off” edge on lateral films [11], aiding in diagnosis. It has been reported that the orientation of the battery’s negative electrode is important in determining esophageal complications [5]. When the negative electrode faces forward, there is a higher likelihood of tracheoesophageal fistula, vocal cord paralysis, and hemorrhage. Conversely, if the negative electrode faces backward, the risk of hemorrhage is higher. However, details regarding the orientation of the battery’s negative electrode were recorded for fewer than 20% of cases in our study, highlighting an area for improvement and further exploration in future research. Enhancing documentation of battery orientation could contribute to more comprehensive understanding and management of BB ingestion cases.

Current recommendations emphasize the importance of removing BBs within 2 h of ingestion due to the serious injuries [2, 10]. However, in routine clinical practice, it can be challenging to perform esophagoscopy within such a short timeframe. Our study revealed that BB removal was often delayed due to the need for transfer to a specialized pediatric hospital facility, resulting in a median time from ingestion to hospital admission of 3 h (range: 0.5 h to 3 months). To address this challenge, we have for the first time adopted an aggressive strategy of sending patients directly to the operating room without preoperative examination once the diagnosis was confirmed. While this approach carries a risk of pulmonary aspiration, it is outweighed by the severity of the BB hazard and the benefits of first-aid fast track strategies [12]. The choice between performing rigid esophagoscopy (RE) or flexible esophagoscopy (FE) depends on the practices and experience of the medical institution or physician. Systematic reviews and meta-analyses have shown that the safety and efficacy of both methods are comparable [13, 14]. The use of a Foley catheter for esophageal BB removal is not recommended due to its blinded technique, which prevents assessment of the periesophageal mucosa and may potentially increase the risk of airway injury, vomiting, and esophageal perforation.

The European Society for Paediatric Gastroenterology Hepatology and Nutrition (ESPGHAN) guidelines in 2021 recommend administering honey (1 g) and/or sucralfate suspension (10mL) at home for cases of BB ingestion, as esophageal perforation is less likely to occur within the first 12 h after ingestion [10]. However, this advice is applicable only in cases where there is a history of witnessed BB ingestion and in children older than 1 year old, as there is a small risk for infant (< 1 year old) botulism after honey intake. It’s important to note that referring institutions may not provide honey or sucralfate while awaiting transfer due to lack of experience or unavailability of these substances.

Numerous animal experiments have explored therapeutic approaches to mitigate rapid tissue damage following BB ingestion. Previous research demonstrated that using an acidic solution, such as acetic acid, in cadaveric piglet esophageal tissue could help minimize esophageal damage [5]. Irrigation with 0.25% acetic acid after BB removal was shown to neutralize tissue pH from 12 to 6 [15]. Additionally, various acidic drinks, including coke, orange juice, honey, and sucralfate, were found to decrease pH levels and reduce esophageal injury. Based on these findings, some authors suggest the potential benefits of administering a weakly acidic solution orally immediately after ingestion to reduce esophageal damage before emergent endoscopic BB removal. However, this intervention poses several controversial issues that require further research. For instance, it may not be safe to administer anything orally if esophageal perforation or tracheoesophageal fistula is suspected, as it could increase the risk of tracheal aspiration due to the BB being impacted in the esophagus. Overall, while these experimental findings are promising, additional research is needed to address safety concerns and determine the optimal timing and method of administration for acidic solutions in cases of BB impactions.

Litovitz [3] reported that serious complications such as esophageal stenosis, esophageal perforation, and tracheoesophageal fistula can occur in children with BB impactions. Vocal cord paralysis, hemorrhage, mediastinitis, periesophageal abscess, and pneumothorax have also been reported as severe complications, although their incidence is very rare. The factors contributing to complications in BB impactions are complex. Fatal cases reported by the National Poison Data System identified the size of BB (≥ 2 cm) as the most important predictor, followed by age under 5 years and ingestion of more than one battery [3]. A recent study has identified age younger than 3 years, unwitnessed ingestion, and the size of BB (≥ 2 cm) as significant predictors of severe complications [16]. However, the location of the esophageal battery and the duration of impaction were not significant predictors in this research. In our study, complications were significantly associated with a longer duration of foreign body impactions, BB diameter ≥ 2 cm, and unwitnessed ingestion. We found that the BB diameters ranged from 1 cm to 2.5 cm, with the majority (80.9%) being larger than 2 cm, which are more prone to lodging in the esophagus of children and causing extensive esophageal damage. Additionally, the presence of fever and cough may indicate periesophageal inflammation or tracheal damage, further emphasizing the severity of the condition.

After surgery, patients are admitted to the hospital and fed via gastric tube while also receiving treatment with acid-inhibiting agents. An esophagogram can be administered 1 to 2 days after removal, and a liquid oral diet can be initiated if there are no signs of esophageal perforation [10]. In complicated cases, the period of gastric intubation should be extended until the patients’ condition stabilized. Antibiotics to prevent periesophageal inflammation should be considered in patients with severe damage, perforation, and fever. Esophageal imaging inspection (CT or MRI) is performed 1 week after BB removal in patients with significant injuries to confirm the tissue integrity between the esophagus and great vessels [3, 10]. Studies have reported that most children with esophageal perforation are diagnosed within 1 week after surgery. However, some complications, such as esophageal stenosis, may occur within a few weeks after removal, or even appear after 6 to 8 months [17]. In our cohort, most complications were diagnosed intraoperatively or within a week after surgery. However, one case of tracheoesophageal fistula occurred 1 month after removal, highlighting the importance of vigilance for delayed complications and the need to extend the follow-up time accordingly.

Esophageal stenosis and perforation were the most common complications observed in our study, and tracheoesophageal fistula should be considered when symptoms such as fever and cough occur after the operation. Fortunately, most children with complications can heal spontaneously without surgery [9, 18, 19]. Following the operation, patients were fed via gastric tube, and active anti-infection, acid inhibition, and symptomatic treatment were administered for a week. In two cases complicated by esophageal cicatricial stenosis, balloon esophageal dilatation proved to be effective, leading to cure within 1-month post-operation. Another case, tormented with tracheoesophageal fistula, recovered after the insertion of self-expandable metallic stents within 2 months after BB removal. In our treatment experience, when esophageal stenosis or perforation occurs after the operation, non-surgical treatment should be prioritized. Revaluation can be administered after 1 month of dynamic observation, and surgical intervention will be considered if the patient does not recover spontaneously.

Bleeding is the most dangerous complication of BB impactions, especially when the battery is located in the middle esophagus surrounded by great vessels. Fatal outcomes may result from massive hemorrhage due to fistula formation to the great vessels, such as aortoesophageal fistula, or suffocation secondary to blood aspiration [10]. In such cases, consultation with interventional doctors and cardiothoracic surgeons is essential. Esophageal imaging inspection (CT or MRI) should be performed to confirm the tissue between the esophagus and great vessels. Additionally, arteriogram allows direct visualization of the position of the battery and aorta. A joint approach involving the departments of cardiothoracic surgery and intervention may be necessary in massive hemorrhage cases. In our study, one child experienced intraoperative bleeding, while another child had bleeding 2 days after removal. Both patients were successfully treated after emergency exploration and hemostasis.

## Conclusion

The most effective way to prevent these injuries is to strengthen family education regarding the safe use, storage, and disposal of button batteries. Manufacturers should consider producing smaller diameter batteries to reduce the risk of foreign body impactions. Additionally, government regulatory institutions should be established to oversee and address adverse events related to battery ingestion. Primary health care institutions should be vigilant in identifying BB impactions in children and promptly refer them to specialized pediatric hospital facilities. We recommend performing a first-aid fast track rigid esophagoscopy under endotracheal intubation anesthesia within half an hour of establishing the diagnosis, without waiting for preoperative examinations or fasting time. Early intervention is crucial in reducing the occurrence of complications with BB impactions in children.

## Data Availability

The datasets used and analyzed during the current study are available from the corresponding author on reasonable request.

## Abbreviations

EFB: Esophageal foreign body
BB: Button battery
RE: Rigid esophagoscopy
FE: Flexible esophagoscopy
CT: Computed tomography
MRI: Magnetic resonance imaging
